# Do Mast Cells Contribute to the Antifungal Host Defense?

**DOI:** 10.3390/cells10102510

**Published:** 2021-09-22

**Authors:** Paulina Żelechowska, Joanna Pastwińska, Ewa Brzezińska-Błaszczyk, Justyna Agier

**Affiliations:** 1 Department of Microbiology and Experimental Immunology, Faculty of Health Sciences, Medical University of Lodz, Pomorska 251, 92-213 Lodz, Poland; joanna.pastwinska@stud.umed.lodz.pl (J.P.); ewab@csk.umed.lodz.pl (E.B.-B.); justyna.agier@umed.lodz.pl (J.A.); 2 Laboratory of Epigenetics, Institute of Medical Biology, Polish Academy of Sciences, Lodowa 106, 93-232 Lodz, Poland

**Keywords:** mast cell, fungi, fungal infection, host defense, pathogen-associated molecular pattern

## Abstract

The fungal kingdom includes a group of microorganisms that are widely distributed in the environment, and therefore the exposure to them is almost constant. Furthermore, fungal components of the microbiome, i.e., mycobiome, could serve as a reservoir of potentially opportunistic pathogens. Despite close encounters with fungi, defense mechanisms that develop during fungal infections remain unexplored. The strategic location of mast cells (MCs) close to the external environment places them among the first cells to encounter pathogens along with the other innate immune cells. MCs are directly involved in the host defense through the ability to destroy pathogens or indirectly by activating other immune cells. Most available data present MCs’ involvement in antibacterial, antiviral, or antiparasitic defense mechanisms. However, less is known about their contribution in defense mechanisms against fungi. MCs may support immune responses to fungi or their specific molecules through initiated degranulation, synthesis and release of cytokines, chemokines, mediators, and generation of reactive oxygen species (ROS), as well as immune cells’ recruitment, phagocytosis, or provision of extracellular DNA traps. This review summarizes current knowledge on host defense mechanisms against fungi and MCs’ involvement in those processes. It also describes the effects of fungi or fungus-derived constituents on MCs’ activity.

## 1. Introduction

**Figure 1:**
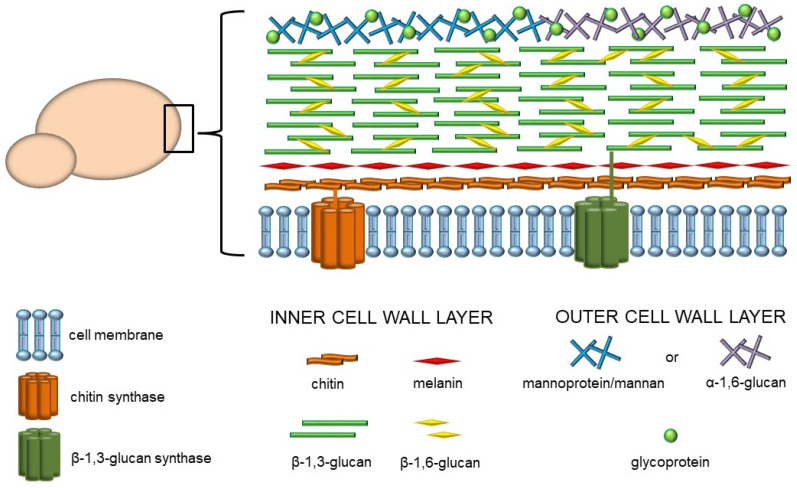
Structural organization and composition of the fungal cell wall.

The prevalence of fungal infections presents a global health issue and has been escalating in recent years. Due to the common multidrug resistance as well as lack of effective antifungal remedies, fungal infections have become increasingly difficult to treat. Moreover, in contrast to bacterial or viral infections, a successful vaccine against pathogenic fungi has not yet been developed [1,2]. Since fungi are ubiquitous in the environment, we are constantly and directly exposed to them on many levels. Furthermore, fungi form an integral part of the microbiome, which plays a role as a reservoir for opportunistic pathogens. The mycobiome maintains body homeostasis and influences the immunological responsiveness in the host organism. However, it has been reported that altered immune system function or mycobiome composition play an important role in several human diseases [3]. Despite the close encounters with fungi, the host defense mechanisms against various fungal pathogens are not fully known. One of the significant challenges is the unique structure of the fungi cell wall, which is the crucial element involved in the interaction with the host. In general, the fungal cell wall is a rigid structure composed of various types of linear and branched polysaccharides, glycoproteins, proteins, lipids, and other components. These components may be organized into at least two layers (Figure 1). The inner layer of most pathogenic fungi consists of a core of glycans, including chitin, which is located closest to the plasma membrane, and β-1,3- and β-1,6-glucans adjacent to the chitin fibers. It has been indicated that 50–60% of the dry weight of this structure is made up of glucan with β-1,3-glycosidic linkages. Many fungi have an inner cell wall layer that also contains complex polymerized and/or indolic compounds called melanins. On the other hand, the composition of the outer layer may vary among fungal species, but generally, it is composed of α-1,3-glucans or homopolymers of mannose, such as mannans and mannoproteins [4].

Host defense against various pathogens, primarily bacteria and viruses, involves the orchestration of first-line innate immune responses and adaptive immunity mechanisms. The course of these processes depends to a large extent on the involvement of mast cells (MCs). However, no sufficient data have been found on MCs’ contribution to defense against fungi. This review summarizes current knowledge of host defense mechanisms against fungi and MCs’ involvement in those processes. It also provides insight into the effects of fungi or specific fungus-derived constituents on MCs’ activity.

## 2. Microscopic Fungi and the Immune System

Microscopic fungi and the substances they produce are quite common in the environment but also constitute the natural microbiota that colonizes the mucous membranes and the skin of living organisms, e.g., *Candida* spp. Under certain conditions, mainly due to tissue homeostasis disruption, infection with external pathogenic microorganisms and commensal species of fungi may occur. However, despite daily exposure to pathogens, a healthy organism usually resists all infections. This is due to the development of numerous sophisticated defense mechanisms, whose role is to recognize the threat and eliminate it [5,6]. The skin and mucous membranes overlining different surfaces in the body, e.g., in the respiratory system and gastrointestinal and genitourinary tracts, constitute a natural physical barrier limiting the penetration of microorganisms. Moreover, natural antimicrobial substances produced in the organism, such as β-defensins, cathelicidins, surfactant protein A (SP-A), SP-D, lysozyme, lactoferrin, and mucins [5,7,8] as well as physiological microbiota provide a barrier that prevents the multiplication of foreign fungi [7]. In spite of a fungal pathogen defeating the physical barriers, further obstacles await it. These include the expression of various extra- and intracellular receptors specialized in pathogen recognition or secreted humoral factors [7,9].

Almost all types of immune cells are involved in antifungal defense, as each has different functions, complementing each other and creating an unbreakable network of interconnections. Among them, we can distinguish phagocytic cells such as neutrophils, monocytes and their tissue counterparts, and macrophages, but also dendritic cells (DCs), natural killer cells (NKs), B cells, various populations of T lymphocytes, or epithelial and endothelial cells. Numerous defensive substances are produced by these cells in order to combat the ongoing fungal infection, mostly indirectly through induction of chemotaxis and activation of other immune cells or by initiating various defense processes. Depending on the fungal etiological agent, they may play a major or minor role in recognizing and eliminating these pathogens. Monocytes infiltrate infected tissues and transform into macrophages which, apart from phagocytosing fungal pathogens, secrete inflammatory mediators to recruit and activate other immune cells, including neutrophils [6,7]. Macrophages particularly target the elimination of *Cryptococcus* spp. and *Pneumocystis* spp. Moreover, in the case of alveolar macrophages, they also target *Aspergillus fumigatus.* Despite coming from macrophages, neutrophils also play an essential role in the prevention of *C. albicans* and *A. fumigatus*. Therefore, neutropenia strongly predisposes to, e.g., *Candida* infections. In addition, neutrophils regulate the generation of reactive oxygen species’ (ROS) as well as use non-oxidative mechanisms in fungus elimination. Hence, they are responsible for the release of antimicrobial substances, such as β-defensins, lysozyme, lactoferrin, elastase, gelatinases, and cathepsin G. For example, elastase regulates the formation of neutrophil extracellular traps (NETs) composed of neutrophil DNA, especially useful in immobilization and neutralization of fungal hyphae due to their size, which prevents phagocytosis [6,7,10]. On the other hand, dendritic cells, after contact with *Candida* spp., release interferon (IFN)-β in spleen tyrosine kinase (SYK)-dependent and IFN regulatory factor 5 (IRF5)-dependent pathways, apart from phagocytosis and presentation of antigens. Further presentation of antigens leads to the activation of T helper (Th) cells, e.g., Th17, which, through the production of IL-17 and IL-22, contribute to the recruitment of neutrophils as well as enhance the release of β-defensins by epithelial cells. In addition, Th17 cells, through the production of IL-17A, appear to play a vital role in the immune response against *C. albicans* [6,7,11]. CD4^+^ and CD8^+^ T cells, including Th1, are also involved in response to fungal infection, specifically to *Cryptococcus neoformans* in the central nervous system (CNS). It is suggested that Th2 cells may be involved in infections with dermatophytes, such as *Trichophyton rubrum*, due to the presence of IgE and IgG4 antigens of *T. rubrum* in the serum. As a result, the cytokines IL-4, IL-5, and IL-10 are produced [12]. Often underestimated in immune defense, platelets are also involved in the anti-*Candida* response by stimulating the synthesis of antifungal CCL5 and platelet factor 4 (PF4). Antibodies, whose role is to opsonize fungi, prevent their adhesion, or neutralize toxins, are a unique product of B cells [6,7].

Before the immune system can fight the pathogen, it must first recognize the threat in order to send the best-adapted units to the rescue. To this end, pattern recognition receptors (PRRs) serve as signaling proteins that can recognize microbial-associated molecular patterns (MAMPs) specific for various microorganisms and damage-associated molecular patterns (DAMPs), which are released by damaged cells or tissues of the host. Among them, we can distinguish Toll-like receptors (TLRs), C-type lectin receptors (CLRs), NOD-like receptors (NLRs), and RIG-I-like receptors (RLRs) [5,6,9,10,13]. Some of the more important TLRs are TLR2 and TLR4, which are stimulated by *Candida* spp. and *Aspergillus* spp. Surface substances present on these fungi, such as zymosan, phospholipomannan, and glucuronoxylomannan (GXM) stimulate TLR2. In contrast, GXM and O-linked mannan are the most common ligands for TLR4 [5,6,9]. It is notable that the signaling pathway induced by TLR4 leads to the generation of a more vigorous response associated with the activity of pro-inflammatory Th1 cells than in the case of TLR2 connected to the predominance of anti-inflammatory Th2 cells and regulatory T cells (Treg) [9,10]. Other important TLRs are TLR3 (with double-stranded RNA from conidia as a ligand) inducing IFN-γ production, TLR6 (with zymosan as a ligand) regulating IL-17 and IL-23 production, and TLR9 recognizing chitin and unmethylated CpG motifs from fungal DNA [5,6,9,10,13]. Dectin-1, a known CLR, can bind β-glucans (including zymosan) present in the cell walls of, e.g., *Candida* spp. and *Aspergillus* spp. [5,6,9,10,13,14,15]. It also has a function in regulating the formation of NETs, mainly to prevent their uncontrolled formation [6,10,15]. Moreover, TLR2 and Dectin-1 collaboration leads to increased stimulation of the immune system by the release of the pro-inflammatory cytokines. Activation of these receptors may also signal the immune cells to begin phagocytosis [5,6,9,13]. Another CLR is the mannose receptor (MR), which, apart from mannans and mannoproteins, also recognize the glycoprotein A (gpA) complex [5,6,9,10,14,15]. Specifically, macrophage MR can identify *Candida auris* mannoproteins, which stimulate immune response more than *C. albicans* [6,15]. Dectin-2 and Dectin-3, boosted by *Candida glabrata* α-mannan and DC-specific intercellular adhesion molecule-3-grabbing non-integrin (DC-SIGN), as well as macrophage-inducible Ca^2+^-dependent lectin receptor (Mincle), all responsive to *C. albicans* infection and the presence of mannans and mannoproteins, also belong to the CLR family. Mincle activation is associated with tumor necrosis factor (TNF) production, while DC-SIGN regulates Th cell activation and differentiation [5,6,9,14,15]. TLR and CLR signaling pathways are often involved in signal transduction by the CARD9 adapter protein, a crucial component of NFκB and Syk pathways, leading to the secretion of TNF, IL-1β, IL-6, IL-12, and granulocyte–macrophage colony-stimulating factor (GM-CSF). Lack of CARD9 predisposes the body to increased fungal infections [6,14]. MyD88 is also exemplified as a molecule in the TLR signaling pathway that plays a vital role in maintaining fungal infections [6,10,13,14]. Slightly less is known about the contribution of NLRs and RLRs to the antifungal response. However, from the available data, it can be concluded that melanoma differentiation-associated protein 5 (MDA5), a receptor belonging to the RLR family, recognizes *Candida* spp. RNA. As for NLRs, there are known representatives of NOD1 and NOD2, which activate the NFκB pathway through receptor-interacting protein 2 (RIP2). Chitin is a well-known ligand for NOD2. Moreover, within this family, proteins such as NLRP1-14 are known to form complexes called inflammasomes. There are indications that *A. fumigatus* can activate the formation of these complexes, which leads to the formation of active forms of IL-1β and IL-18 from inactive precursors [5,6,9,10,13,14,15].

Just as the immune system has successfully adapted to kill microbes, pathogens have developed mechanisms that enable them to survive, making these strategies no less critical than virulence factors. These processes include the production of immune-suppressing particles, shielding of stimulatory MAMPs, adapting the composition and organization of the cell wall, biofilm formation, inducing an anti-inflammatory response rather than a pro-inflammatory, or dimorphic capacity [9,16]. Many fungi, including *Metarhizium anisopliae*, can produce toxic peptides, i.e., destruxins, that suppress the production of antimicrobial peptides in *Drosophila melanogaster*, which is a model for human immune responses in the transduction of key signals [17]. *Candida* spp., in response to the prevention of adhesion to the skin, developed adhesins, proteins related to β-glucan that also enable the formation of biofilms. Some fungi, including *C. albicans*, can change their phenotype, thanks to which they can hide the molecules detected by PRRs, such as β-glucan. Another way to hide MAMPs is to get rid of them, as in the case of *Coccidioides posadasii*, which uses a secreted metalloproteinase to digest spherule outer wall glycoprotein (SOWgp). *Pneumocystis* spp. developed a mechanism involving the release of gpA that blocks MR, thus protecting the pathogen from opsonization. Pathogenic fungi, such as *C. albicans* and *C. neoformans*, modulate the immune response in the anti-inflammatory direction, mainly by TLR2 stimulation and subsequent Th2 recruitment. An equally clever way to avoid phagocytosis is the ability to enter host cells such as epithelial and endothelial cells, as in the case of *C. albicans*, *A. fumigatus*, and *C. neoformans* [9,16].

## 3. How Do MCs Contribute to the Host Defense?

**Figure 2:**
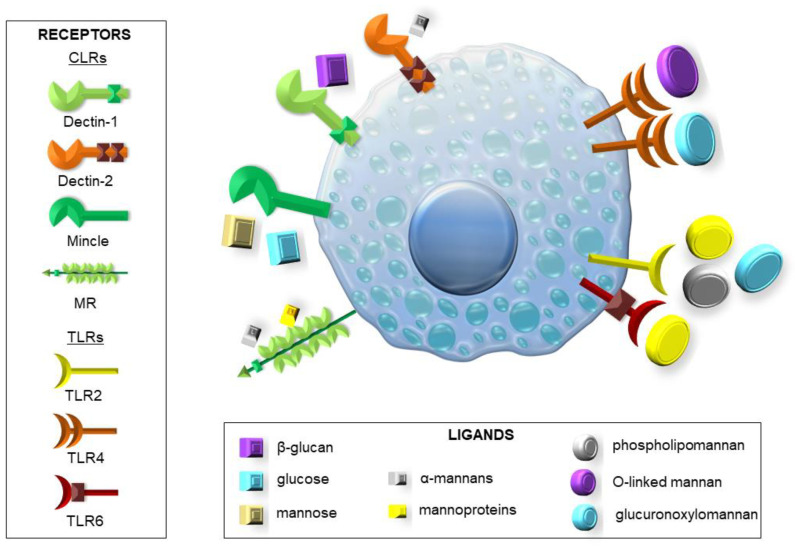
Expression of PRRs involved in sensing different fungal components on MCs.

MCs play a pivotal role in the host defense against pathogenic microorganisms for several reasons. First of all, MCs’ strategic position at the host–external environment interfaces, i.e., in the subepithelial layers of the skin, the respiratory system, or in the gastrointestinal and genitourinary tracts, means that they are among the first cell population to interact with invading microbes along with other innate immune cells, such as epithelial cells, and trigger a response against them. Moreover, it is well known that MCs may initiate and combat the clearance of pathogens by several mechanisms of action [18,19,20]. MCs possess an array of bioactive substances, which affect all stages of inflammation during infection, including its initiation, maintenance, and even resolution. They include granule-associated preformed mediators (e.g., histamine, tryptase, chymase, carboxypeptidases, metalloproteinases, proteoglycans), de novo-produced eicosanoid metabolites (e.g., leukotrienes (LTs), prostaglandins, thromboxanes), as well as many newly synthesized cytokines/chemokines [21,22,23,24,25]. MC activity against pathogens also involves the release of some antimicrobial peptides and the production of ROS. Moreover, these cells can engulf invading microbes via phagocytosis and kill them through oxidative and non-oxidative systems [18,19]. Another described strategy used by MCs to destroy microorganisms is through extracellular traps (MCETs) composed of DNA, histones, and granule proteins [26]. The relevance of MCETs has been documented in antibacterial, antifungal, or antiparasitic host defense [27,28]. Following phagocytosis, MCs may process pathogen antigens for presentation through class I and II MHC molecules, which leads to the development of adaptive antimicrobial immunity [29]. As MCs express PRRs, they may act as effectors of host defense through their ability to detect various MAMPs or endogenous DAMPs released in response to infection. The available data indicate that among PRRs expressed on MCs, there are representatives from TLRs, RLRs, and NLRs, as well as CLRs [30]. It has been reported that MCs can recognize bacterium-associated molecules, such as lipopolysaccharide (LPS), lipoteichoic acid (LTA), or peptidoglycan (PGN) mainly through TLRs but also via some NLRs. In turn, specific TLRs and RLRs are involved in MC response to viral dsRNA, ssRNA, or envelope proteins. Among PRRs expressed on MCs, there are also molecules from the CLR group or some TLRs, which may sense different fungal components (Figure 2) [30]. However, little information exists concerning MC involvement in antifungal host defense, and the function of MCs in fungal infections is not precisely defined [31,32].

## 4. Expression of PRRs Involved in Fungus Recognition in MCs

It is well known that CLR family members are strongly involved in recognizing fungi and their components [33]. However, data showing the expression of these receptors within MCs are still limited. Among investigated receptors from the CLR group, the most presented reports concerned Dectin-1 primarily. Only a handful of studies revealed the expression of Dectin-2, MR, or Mincle within MCs, whereas Dectin-3’s presence on these cells has not been investigated yet. The constitutive expression of Dectin-1 has been confirmed in bone marrow-derived MCs (BMMCs) [34], cord blood-derived MCs (CBMCs) [35], a human leukemic cell line (KU-812) that displays enhanced tryptase expression [35], the rat basophilic leukemia clone 2H3 cell line (RBL-2H3), i.e., mucosal-like MCs [36], and tryptase-positive progenitor-derived MCs [37], both at mRNA and protein levels. We have recently documented that rat connective tissue type MCs, i.e., peritoneal MCs (PMCs), also constitutively express Dectin-1 and Dectin-2 mRNA and protein [38,39]. To date, the expression of mRNA and protein of Mincle has been demonstrated only in RBL-2H3 cells [40] and tryptase-positive progenitor-derived MCs [37]. The protein expression of MR was documented in murine BMMCs and PMCs [41].

Expression of all known members from the TLR family has been confirmed in a wide range of MC types and cell lines. However, as mentioned earlier, it has been established that only certain TLRs participate in fungus sensing. Among them, the most important for fungus detection seems to be TLR4. The mRNA or protein expression of TLR4 was documented in a mouse MC line (MC/9) [42], in MC lines that closely resemble primary human MCs, i.e., a human MC line (HMC-1) and Laboratory of Allergic Diseases 2 (LAD2) cells [43], murine BMMCs [44], murine fetal skin-derived cultured MCs (FSMCs) that exhibit important features of connective tissue type MCs [44], and CBMCs [45], as well as rat and murine PMCs [44,46]. TLR2 is another well-characterized PRR participating in fungus recognition. Available data indicate that mRNA and/or protein expression of TLR2 are found in MC/9 [42], HMC-1 and LAD2 cells [43,47], murine BMMCs and FSMCs [44,48], CBMCs [45], and in mature rat PMCs [46]. Since TLR2 forms heterodimers with TLR6 to recognize fungal components, it should be stressed that its expression has also been documented on different MC types, including MC/9 [42], HMC-1, LAD2 cells [49], human cultured MCs (HCMCs) [49], FSMCs and BMMCs [44], CBMCs [45], and rat PMCs [46].

An interesting observation is that fungi or fungus-derived constituents may modulate the expression of some PRRs in MCs. Ribbing et al. [37] documented that the stimulation of tryptase-positive progenitor-derived MCs with *Malasezzia sympodialis* extract or after IgE receptor cross-linking resulted in an increased expression of Mincle mRNA. *Saccharomyces cerevisiae*-derived zymosan, i.e., β-1,3-glucan containing mannan particles, significantly upregulates surface expression of the Dectin-1 receptor in murine BMMCs [34]. Only a few reports exist regarding the effect of various endogenous factors on PRRs involved in fungus recognition in MCs. Our observations provided evidence that IL-6 treatment of PMCs induces an increase in TLR4 expression, whereas exposure of those cells to CCL5 results in decreased expression of both TLR2 and TLR4 [50]. Okumura et al. [51] found that IFN-γ upregulates TLR4 expression on human peripheral blood-derived MCs and Yang et al. [52] demonstrated that IL-12 induces a significant increase in the expression of TLR2 and TLR4 mRNAs and proteins in the P815 cell line. Our recent data have revealed that one of the well-known antimicrobial peptides, i.e., cathelicidin LL-37, increases TLR2 and TLR4 expression in the PMCs [53]. Similar observations were made by Yoshioka and colleagues [54] as they observed augmented TLR4 expression in LAD2 cells in response to LL-37.

## 5. Fungi Affect MC Activity

### 5.1. Effect of Fungal Cells on MCs

Evidence indicates that fungi directly induce MC activity in vitro, and most of the available data concern their effect on the generation and/or release of different mediators from MCs (Table 1). It has been documented that some species of fungi stimulate MCs to release preformed mediators. They also promote the production of a variety of factors, cytokines, and chemokines, including those which are known for their potent inflammatory and antifungal activities. Yeasts, hyphae, and the cell wall fraction of *C. albicans* activate murine BMMCs, HMC-1, and RBL-2H3 cells as well as rat PMCs, thus resulting in degranulation and β-hexosaminidase release [55,56,57]. Additionally, Nieto-Patlán and co-workers [55] evaluated BMMC degranulation by the exposure of CD107a on the cell surface after MC activation. They reported that the percentage of BMMCs positive for CD107a increases after stimulation with *C. albicans* hyphae or yeasts compared to non-stimulated cells [55].

*Paracoccidioides brasiliensis* cells and mature *A. fumigatus* hyphae, but not conidia or immature hyphae, also induce RBL-2H3 cell degranulation and β-hexosaminidase secretion [58,63]. Moreover, *C. neoformans* cells trigger CBMCs and HMC-1 to release β-hexosaminidase and tryptase [61]. Conversely, treatment of murine BMMCs with *M. sympodialis* extract as well as incubation of those cells and rat PMCs with conidia or cells of *Sporothrix schenckii* yeast does not induce MC degranulation [62,64,65]. BMMCs do not degranulate in response to *S. schenckii* as assessed by β-hexosaminidase release and CD63 surface expression by flow cytometry [66]. Such ambiguous observations suggest that conventional MC degranulation may not be a prerequisite for preformed MC mediators to exert a protective effect during fungal infections. It was also observed that *M. sympodialis* or *S. schenckii* activate BMMCs and/or rat connective tissue type PMCs to release robust pro-inflammatory mediators, i.e., cysteinyl LTs (cysLTs) and cytokines, including TNF and IL-6 [62,64,66]. *M. sympodialis* extract enhances the release of cysLTs from IgE-sensitized BMMCs and induces their degranulation and CCL2 production [62]. It was also noted that peripheral blood MCs obtained from atopic eczema patients release increased amounts of IL-6 in response to *M. sympodialis* [37]. This report suggests that MC-derived de novo-synthesized cytokines may contribute to the skin inflammation in atopic eczema during fungal infection. Interestingly, it has been indicated that MC tryptase may serve as a potential biomarker of the *A. fumigatus*–host interaction since production of this protease is decreased in patients displaying specific IgE to *A. fumigatus* compared to patients without detectable IgE to this fungus [67]. It is well known that MCs play a vital role in extracellular matrix remodeling/degradation through the release of preformed enzymes, such as tryptase, chymase, or carboxypeptidases. MC-derived proteases improve fibroblast and epithelial cell proliferation and leukocyte recruitment. They also induce angiogenesis as well as collagen synthesis in damaged tissue [20]. Therefore, it may be suggested that in a specific tissue microenvironment, i.e., in the site of pathogen contact, MCs promote wound healing or fibrotic events [68]. Both yeasts and hyphae of *C. albicans* stimulate murine BMMCs to synthesize pro-inflammatory cytokines, including TNF, IL-6, IL-13, chemokines CCL3, and CCL4, but also anti-inflammatory IL-10 [55,60]. Moreover, only yeasts of *C. albicans* activate BMMCs to produce and release IL-1β, which exerts potent pro-inflammatory activity [55]. Upon *C. albicans* stimulation, HMC-1 cells secrete interleukin-1 receptor antagonist (IL-1Ra), IL-16, and migration inhibitory factor (MIF) [57]. Renga et al. [59] reported that stromal MCs produce higher concentrations of transforming growth factor β (TGF-β) and IL-10 than mucosal MCs in response to hyphae of *C. albicans*. The authors suggest that *C. albicans* exploits MC functional versatility at mucosal surfaces to contribute to local damage and inflammation or protection [59]. Interestingly, HMC-1 cells infected with *C. albicans* secrete significant amounts of CXCL8 [57], a chemokine possessing a solid chemotactic activity for neutrophils. Of note, another opportunistic pathogen, *Pneumocystis jirovecii,* is an atypical fungus with lung tropism that causes pneumonia in immunosuppressed individuals, such as HIV infection. Based on the available data, it can be speculated that MCs modulate the duration of opportunistic fungal infections through the release of the mediators. It has been reported that *P. jirovecii*-infected patients present high expression levels of IL-2, IL-4, IL-10, and IL-13 compared to healthy subjects [69]. It is worth noting that MCs are an important source of these multifunctional cytokines. Interestingly, an increased prevalence of *P. jirovecii* colonization has also been observed in patients with chronic obstructive pulmonary disease, in which MCs seem to be involved [70].

Additionally, it has been established that supernatants from *C. albicans*-infected HMC-1 cells induce migration of neutrophils but not monocytes, supporting the critical role of this chemokine in neutrophil recruitment towards *C. albicans* [57]. In contrast, stimulation with supernatants from *C. neoformans*-infected CBMCs and HMC-1 cells induces migration of macrophages but not neutrophils [61]. It has also been reported that murine BMMCs infected with *C. albicans* augment macrophage crawling ability and promote their chemotaxis [60].

It is worth noting that MCs accumulate in the tumor stroma of a wide range of cancer types. Therefore, the evidence for the role of these cells in shaping cancer and its microenvironment continues to grow [71,72]. Data indicate that fungi are implicated in oncogenesis and the ability to induce inflammation, which may cause cancer. For example, evidence suggests that *C. albicans* infection may be associated with oral cancer development [73]. Bearing in mind an increased MC infiltration into the tumor microenvironment and that MCs are involved in fungal infections, it can be stated that these cells may mediate the development of a tumor or tumors by altering the inflammatory environment. As mentioned earlier, MCs remodel the extracellular matrix during wound healing/tissue repair, but it should be stressed that this function is subverted in tumor growth [20]. Considering that MCs produce pro-fibrotic mediators, such as TGF-β, in response to some fungi or their specific constituents [59], it can also be hypothesized that MCs may contribute to fibrotic lesions during fungal infections.

The available data regarding the mechanisms of direct killing strategies of fungi exerted by MCs are unclear. A few authors indicate that these cells primarily combat the fungi through extracellular mechanisms, whereas others describe their phagocytic capabilities. Indeed, Trevisan et al. [74] reported that rat PMCs have limited capacity to phagocytose *C. albicans*. However, it has been strongly suggested that these cells are able to kill yeasts in the extracellular environment, probably through the fungicidal activity of secreted mediators, including ROS and the enzyme β-hexosaminidase. These observations were confirmed in in vivo studies and in situ findings. Yan et al. [75] created a rat model of acute invasive fungal rhinosinusitis (AIFR) based on *A. fumigatus* infection. The authors revealed that MCs might mainly play a role through degranulation, or the release of cytokines/chemokines involved in immune response instead of direct phagocytosis of fungi during AIFR. They observed that compared to control rats, the total number of MCs is unchanged, but MC degranulation is only found in the infected nasal cavities of AIFR rats [75]. The notion that MCs play a protecting role in fungal infection through their degranulation was also supported by the findings of Xie and co-workers [76]. Histological staining revealed a larger number of degranulated/total MCs in the corneal limbus in a mouse model of *Fusarium* spp. keratitis as compared to normal mice, which results in vasodilation, increased intercellular adhesion molecule-1 (ICAM-1) expression on endothelial cells, and neutrophil infiltration [76]. Furthermore, a significant increase in the number of MCs in the dermis and subcutaneous tissue of rats after inoculation with the metabolic extract of *Fusarium oxysporum* compared to the animals from the control group was documented [77]. In contrast, MCs did not reveal signs of degranulation in response to *S. schenckii* in situ, as assessed by quantitative histomorphometric analysis of infected skin sites of mice [66]. Another important finding noted by Xie et al. [76] was that stabilization of MCs with cromolyn leads to inhibition of MC degranulation, dramatic suppression of vascular dilation/permeability, lower ICAM-1 expression, and markedly reduced neutrophil infiltration, which results in increased fungal growth and higher corneal perforation [76]. To the best of our knowledge, no other studies exist that adequately cover MC stabilization during fungal infection. Therefore, future studies investigating the potential of anti-Siglec-8 or anti-FcεRI therapies in fungal infections would be very interesting to provide new information from a clinical point of view.

Noteworthily, Lopes et al. [57] showed that HMC-1 cells and CBMCs release extracellular structures upon *C. albicans* stimulation. These structures are MCETs, which ensnare pathogens. Additionally, the same authors demonstrated that *C. albicans* yeasts could be internalized by MCs, where they can grow and germinate, leading to disruption of MC membranes and ultimately promoting cell death [57]. MCs also modulate the phagocytic activity of resident macrophages during fungal infections as it has been indicated that uninfected BMMCs inhibit phagocytosis of *C. albicans* by macrophages [60]. In contrast, Pinke et al. [78] reported the ability of murine BMMCs to phagocytose *C. albicans* and produce NO with meaningful participation of both Dectin-1 and TLR2 receptors in this process. It has been also demonstrated that HMC-1 cells are not able to phagocytose capsulated *C. neoformans*. However, in the case of the acapsular mutant strain of this pathogenic fungus, a higher rate of HMC-1 cell phagocytosis and survival of those cells was observed [61].

### 5.2. Effect of Fungus-Derived Molecules on MCs

More detailed studies show that several different fungal components, predominantly derived from the fungal cell wall or fungal metabolites, directly affect MC activity. The first report demonstrating the influence of specific fungal molecules on MCs was presented by Padawer and Fruhman as early as 1968 [79]. By electron microscopy, they observed the phagocytosis of zymosan particles by rat PMCs. Later, an investigation on the effect of fungal molecules on MCs was presented by Nosál and co-workers in 1974 [80]. They found that glycoprotein, a polymeric compound isolated from the strain of *C. albicans* CCY 29-3-109, activates rat MC from peritoneal and pleural cavities to degranulate, as was assessed by histamine release [80]. Several further studies also examined the effect of fungus-derived molecules to initiate MC degranulation. However, similarly to studies with specific fungal species, the results in this context are ambiguous. It was demonstrated that mannan and β-glucan obtained from the *C. albicans* fungal cell wall induce RBL-2H3 cell degranulation and β-hexosaminidase secretion [56]. Interestingly, *Aspergillus oryzae*-derived lectin induces the secretion of β-hexosaminidase from RBL-2H3 sensitized with IgE [81]. Furthermore, mannan and/or zymosan from *S. cerevisiae* stimulates rat PMCs but not CBMCs to degranulate [82,83,84]. In turn, β-glucan derived from *Aureobasidium pullulans* inhibits the degranulation of both RBL-2H3 and BMMCs without causing cytotoxicity towards these cells [85]. Gliotoxin, i.e., metabolite released by pathogenic fungi, also suppresses both FcεRI-dependent and -independent BMMC and RBL-2H3 cell degranulation [86]. Additionally, we and other authors indicated that curdlan, a purified linear β-1,3-glucan produced by the bacterium *Alcaligensis faecalis* and widely used as a model fungal particle, induces PMCs and BMMCs to degranulate as well as histamine and/or β-hexosaminidase release [38,87].

Extensive data show that de novo synthesis of many MC mediators is augmented in response to the fungal cell wall molecules. Our recent findings revealed that mannan derived from *S. cerevisiae* activates rat PMCs to generate potent pro-inflammatory mediators, including cysLTs, TNF, CCL2, and CCL3, as well as immunoregulatory IFN-γ and GM-CSF [82]. In turn, stimulation with zymosan results in an increase of IL-33 mRNA expression [88] as well as LTB_4_, LTC_4_, IL-1β, and GM-CSF synthesis by CBMCs [35,84]. Moreover, our paper reported that PMCs release cysLTs, IFNs, GM-CSF, TNF, and CCL2 in response to zymosan [83]. It has also been demonstrated that PbPga1 protein, a surface antigen of *P. brasiliensis* yeast, induces IL-6 release from RBL-2H3 cells [63]. Curdlan also affects the generation of various mediators from MCs. Kimura et al. [36] documented that this molecule induces mRNA expression of IL-3, IL-4, IL-13, TNF, and CCL2 in RBL-2H3 cells. In turn, we have shown that curdlan activates rat PMCs to synthesize cysLTs, TNF, IFN-α, IFN-γ CCL3, and GM-CSF [38]. Conversely, a fungal metabolite, i.e., gliotoxin, suppresses LTC_4_, IL-13, and TNF release from RBL-2H3 cells [86]. The summary of the influence of fungus-derived molecules on MC mediator synthesis is presented in Table 2.

Considering that ROS are highly toxic to microorganisms, including pathogenic fungi, the information that MCs generate free radicals in response to fungus-derived constituents is highly relevant. It has been established that zymosan from *S. cerevisiae* stimulated ROS and NO generation by murine BMMCs [34,78]. We have recently documented that zymosan and mannan, as well as curdlan, also promote ROS generation by rat PMCs [38,82,83].

Some fungus-associated molecules promote MC chemotactic activity. β-glucans, including zymosan and curdlan, as well as mannan, serve as potent chemoattractants for rat PMCs. Recently, we have shown that these molecules cause migration of rat PMCs, even in the absence of extracellular matrix (ECM) proteins [38,82,83]. It can be therefore stated that MCs accumulate in large numbers at the site of fungal infection. In turn, the accumulation of MCs at the inflamed milieu leads to further exacerbation and amplification of the ongoing inflammatory process.

## 6. MCs and Th17 Cells

MCs may play an essential role in the functions of Th17 lymphocytes, a cell subset that has an influential role in bridging the adaptive and the innate immune response against pathogenic fungi. It should be noted that Th17 cells constitute a source of IL-22 and IL-17, which are essential cytokines for the successful antifungal response [89,90]. Although there is no direct evidence that MCs interact with Th17 cells in host defense mechanisms against fungi, growing evidence demonstrates that MCs are important for Th17 cell differentiation. Therefore, information that stimulation of T cells with supernatant of activated MCs increases the number of IL-17-producing T lymphocytes seems highly relevant [91]. It has also been observed that MC-derived TNF is required to develop IL-17-secreting Th17 cells in a murine model of airway inflammation [92]. IL-33-stimulated MCs also induced Th17 differentiation in eosinophil-deficient mice, leading to neutrophil-dominant airway inflammation [93]. Interestingly, Th17 cells express functional histamine H4 receptor; thus, MC-derived histamine may affect Th17 activity [94]. Indeed, stimulation with histamine or an H4 receptor agonist increases the production of IL-17 by human Th17 cells [94]. Considering that cysLTs and chemokine CCL2 act as chemoattractants for Th17 cells, and MCs are a source of these mediators, it can be assumed that MCs may regulate Th17 infiltration [95,96]. MCs can also promote T cell response in the host defense by modifying the functions of antigen-presenting cells. It has been shown that murine PMCs can undergo an interaction with immature DCs, inducing DC maturation and the release of the T cell-modulating cytokines IFN-γ, IL-2, IL-6, and TGF-β. Such MC-primed DCs subsequently induced T cell proliferation and Th1 and Th17 responses [97]. On the other hand, Th17 lymphocytes may also influence MCs activity. Cho et al. [98] reported that the Th17-mediated inflammatory environment promotes MC accumulation and survival through keratinocyte-derived stem cell factor (SCF).

## 7. Concluding Remarks

**Figure 3:**
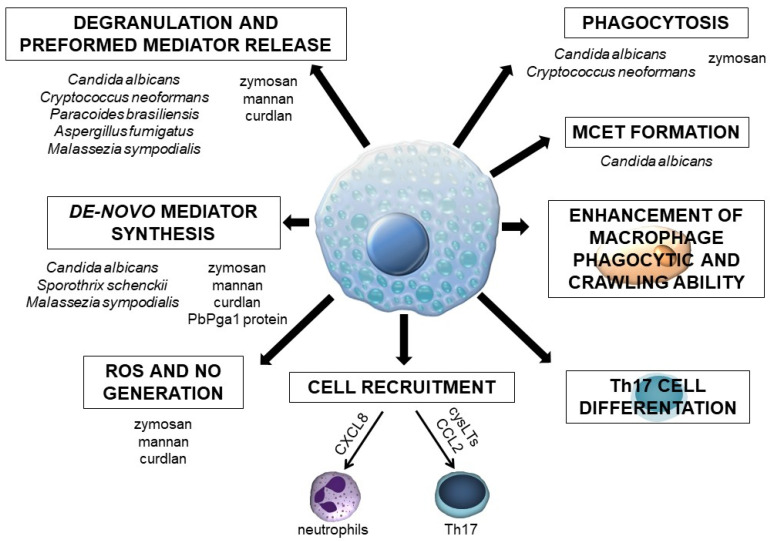
MC activities in response to fungi or fungus-derived molecules.

The currently available data regarding MC involvement in the antifungal host defense are ambiguous, primarily because studies have been carried out on MC types that display various characteristics and express a different array of PRRs. On the other hand, most of the investigations have been conducted with several fungal species that differ in the cell wall structure. Moreover, studies in this field concern whole fungal cells or their specific molecules. Without a doubt, this research area still requires exploration, and it is not yet possible to make definitive statements about the role of MCs in defense mechanisms developing in the course of fungal infection. However, based on the presented facts, it can be assumed that these cells are undoubtedly crucial for initiating a protective inflammatory response to fungal pathogens or their components (Figure 3). To this end, MCs release robust pro-inflammatory mediators, such as histamine and cysLTs, which affect vascular permeability or cell adhesion to the vascular epithelium, enhance phagocyte activity, and generate other factors with pro-inflammatory activities. Moreover, MCs synthesize specific cytokines and chemokines, including those engaged in defense against fungi. They are responsible for activating other immune cells, e.g., fungus-stimulated MCs release CXCL8 accountable for neutrophil recruitment. It should be stressed once again that MCs may contribute to the protection against fungi through forming MCETs. These cells can also eliminate fungi by phagocytosis. Finally, MCs seem necessary for the differentiation and activation of Th17 lymphocytes, a cell population with a leading role in antifungal immunity. The above-mentioned data strongly suggest that MCs act as initiators or regulators of inflammation during fungal infections. Additional support for this comes from some in vivo studies. However, there is a strong need to conduct more studies using MC-deficient experimental models that could provide additional elements for understanding the interaction between MCs and fungi. To date, it has been reported that the inherited MC deficiency in the experimental zymosan-induced peritonitis model significantly impaired the influx of leukocytes and the release of pro-inflammatory cytokines (including IL-1β, IL-6, and TNF), suggesting that MCs initiate a cascade of inflammatory effects [99]. MCs may be involved in the morphine-mediated inhibition of zymosan-induced peritonitis in CBA mice as morphine promotes MC proliferation and/or migration during the early stages of this condition [100].

All these data allow the conclusion that MCs contribute to the control of fungal infections but may also orchestrate mobilization of cells involved in the innate and adaptive immune response. Undoubtedly, there is a strong need to conduct further studies that will help to understand exact MC mechanisms of action in antifungal host defense to develop and implement effective therapeutic strategies.

## Figures and Tables

**Table 1:** The influence of fungi or their cells on MC mediator synthesis.

Fungi	MC Types	Mediators	References
*Aspergillus fumigatus* (mature hyphae)	RBL-2H3	histamine, β-hexosaminidase	[58]
*Candida albicans*	HMC-1	IL-1Ra, IL-16, MIF, CXCL8	[57]
*Candida albicans* (hyphae)	SMCs	TGF-β and IL-10	[59]
*Candida albicans* (yeasts)	BMMCs	IL-1β	[55]
*Candida albicans* (hyphae, yeasts)	BMMCs	TNF, IL-6, IL-10, IL-13, CCL3, CCL4	[55,60]
*Candida albicans* (cell wall, hyphae, yeasts)	BMMCs, HMC-1, PMCs, RBL-2H3	histamine, β-hexosaminidase	[55,56,57]
*Cryptococcus neoformans*	CBMCs, HMC-1	β-hexosaminidase, tryptase	[61]
*Malasezzia sympodialis*	BMMCs, PMCs IgE-sensitized BMMCs PBMCs	cysLTs, TNF, IL-6 histamine, cysLTs, CCL2 IL-6	[37,62]
*Paracoccidioides brasiliensis*	RBL-2H3	histamine, β-hexosaminidase	[63]
*Sporothrix schenckii*	BMMCs, PMCs	cysLTs, TNF, IL-6	[64,65,66]

BMMCs, bone marrow-derived MCs; CBMCs, cord blood-derived MCs; HMC-1, human MC line; PBMCs, peripheral blood MCs; PMCs, peritoneal MCs; SMCs, stromal MCs; RBL-2H3, rat basophilic leukemia clone 2H3.

**Table 2:** The influence of fungus-derived molecules on MC mediator synthesis.

Fungus-Derived Molecules	MC Types	Mediators	References
curdlan (*Alcaligensis faecalis*)	PMCs	histamine, cysLTs, TNF, IFN-α, IFN-γ CCL3, GM-CSF, ROS	[38,87]
	BMMCs	histamine, β-hexosaminidase	
glycoprotein (*Candida albicans*)	PMCs	histamine	[80]
lectin (*Aspergillus oryzae*)	IgE-sensitized RBL-2H3	β-hexosaminidase	[81]
mannan (*Candida albicans*)	RBL-2H3	histamine, β-hexosaminidase	[56,82,83,84]
mannan (*Saccharomyces cerevisiae*)	PMCs	histamine, cysLTs, TNF, CCL2, CCL3, IFN-γ, GM-CSF, ROS	
PbPga1 (*Paracoccidioides brasiliensis*)	RBL-2H3	IL-6	[63]
zymosan (*Saccharomyces cerevisiae*)	BMMCs CBMCs PMCs	ROS, NO LTB_4_, LTC_4_, IL-1β, GM-CSF histamine, cysLTs, TNF, IFNs, GM-CSF, CCL2, ROS	[35,82,83,84,88]
β-glucan (*Candida albicans*)	RBL-2H3	histamine, β-hexosaminidase	[56]

BMMCs, bone marrow-derived MCs; CBMCs, cord blood-derived MCs; PMCs, peritoneal MCs; RBL-2H3, rat basophilic leukemia clone 2H3.

## Data Availability

Not applicable.
